# Direct detection of *falciparum* and non-*falciparum* malaria DNA from a drop of blood with high sensitivity by the dried-LAMP system

**DOI:** 10.1186/s13071-016-1949-8

**Published:** 2017-01-13

**Authors:** Kyoko Hayashida, Kiichi Kajino, Humphrey Simukoko, Martin Simuunza, Joseph Ndebe, Amos Chota, Boniface Namangala, Chihiro Sugimoto

**Affiliations:** 1 Division of Collaboration and Education, Hokkaido University Research Center for Zoonosis Control, Sapporo, 001-0020 Japan; 2 Department of Biomedical Sciences, School of Veterinary Medicine, University of Zambia, P.O. Box 32379, Lusaka, Zambia; 3 Department of Disease Control, School of Veterinary Medicine, University of Zambia, P.O. Box 32379, Lusaka, Zambia; 4 Department of Paraclinical Studies, School of Veterinary Medicine, University of Zambia, P.O. Box 32379, Lusaka, Zambia; 5 Global Station for Zoonosis Control, Global Institution for Collaborative Research and Education (GI-CoRE), Hokkaido University, Sapporo, 001-0020 Japan

## Abstract

**Background:**

Because of the low sensitivity of conventional rapid diagnostic tests (RDTs) for malaria infections, the actual prevalence of the diseases, especially those caused by non-*Plasmodium falciparum* (non-Pf) species, in asymptomatic populations remain less defined in countries lacking in well-equipped facilities for accurate diagnoses. Our direct blood dry LAMP system (CZC-LAMP) was applied to the diagnosis of malaria as simple, rapid and highly sensitive method as an alternative for conventional RDTs in malaria endemic areas where laboratory resources are limited.

**Results:**

LAMP primer sets for mitochondria DNAs of *Plasmodium falciparum* (Pf) and human-infective species other than Pf (non-Pf; *P. vivax*, *P. ovale*, *P. malariae*) were designed and tested by using human blood DNA samples from 74 residents from a malaria endemic area in eastern Zambia. These malaria dry-LAMPs were optimized for field or point-of-care operations, and evaluated in the field at a malaria endemic area in Zambia with 96 human blood samples. To determine the sensitivities and specificities, results obtained by the on-site LAMP diagnosis were compared with those by the nested PCR and nucleotide sequencing of its product. The dry LAMPs showed the sensitivities of 89.7% for Pf and 85.7% for non-Pf, and the specificities of 97.2% for Pf and 100% for non-Pf, with purified blood DNA samples. The direct blood LAMP diagnostic methods, in which 1 μl of anticoagulated blood were used as the template, showed the sensitivities of 98.1% for Pf, 92.1% for non-Pf, and the specificities of 98.1% for Pf, 100% for non-Pf. The prevalences of *P. falciparum*, *P. malariae* and *P. ovale* in the surveyed area were 52.4, 25.3 and 10.6%, respectively, indicating high prevalence of asymptomatic carriers in endemic areas in Zambia.

**Conclusions:**

We have developed new field-applicable malaria diagnostic tests. The malaria CZC-LAMPs showed high sensitivity and specificity to both *P. falciparum* and non*-P. falciparum*. These malaria CZC-LAMPs provide new means for rapid, sensitive and reliable point-of-care diagnosis for low-density malaria infections, and are expected to help update current knowledge of malaria epidemiology, and can contribute to the elimination of malaria from endemic areas.

**Electronic supplementary material:**

The online version of this article (doi:10.1186/s13071-016-1949-8) contains supplementary material, which is available to authorized users.

## Background

Malaria is one of the commonest and most important infectious diseases in tropical and sub-tropical countries. In sub-Saharan Africa, *Plasmodium falciparum* is thought to be the most prevalent among *Plasmodium* species. Although *P. falciparum* has been well investigated epidemiologically, non-*P. falciparum* malaria infections have been rather neglected because of their less severe clinical symptoms and difficulties of diagnosis. Therefore, the endemicity of *P. vivax* has only been reported recently [1], and actual distributions of *P. malariae* and *P. ovale* are still unknown [2].

Recently it turned out to be clear that the duration of infections with both *P. falciparum* and *P. malariae* is quite longer than previously estimated, despite the fact that these two species do not turn into hypnozoite, the dormant liver stage usually observed in *P. vivax* and *P. ovale* infections [3]. During chronic infection phase of malaria, parasitemia always are kept at low density. In high endemic area of malaria, asymptomatic *P. falciparum*-infected individuals with low-density parasitemia that is undetectable by the conventional rapid diagnostic tests (RDT) including light microscopy test or immunochromatographic test (ICT), are very common. In view of the fact that even the density of parasitemia is very low, it is possible to transmit malaria gametocytes into *Anopheles* mosquitos, part of which become infective to human. Consequently, asymptomatic *P. falciparum* or *P. malariae* carrier could be the reservoir for malaria infection. Therefore, highly sensitive diagnostic tools which are able to detect low-density parasitemia are essential for elimination of malaria.

Molecular diagnostic tests are highly sensitive methods to detect malaria infection even at very low parasitemia levels. For instance, polymerase chain reaction (PCR) amplification is one of the most sensitive and reliable methods to detect malaria parasite DNA. Compared to RDTs, PCR requires an expensive equipment such as a thermal-cycler, and well-equipped and clean laboratory conditions. Nucleic acid extraction tools from blood samples are sometime required for PCR. Therefore, PCR is not suitable for the use in resource-poor settings where malaria is endemic. Loop-mediated isothermal amplification (LAMP) is an alternative molecular method which requires neither expensive machines nor clean laboratory [4]. Although LAMP is more user-friendly, it still needs burdensome blood sample preparation.

Previously, we have developed a direct blood dry LAMP diagnostic system, named CZC-LAMP, for detection of human African trypanosomiasis (HAT) [5]. As is the case with conventional RDT, a drop of blood obtained by pricking a finger or an earlobe is sufficient and able to be used directly for the CZC-LAMP. In this study, we applied this system to develop a new easy-to-use RDT that is able to differentiate *P. falciparum* and non-*P. falciparum* even in cases with low density infections.

## Methods

### LAMP primers

**Fig. 1:**
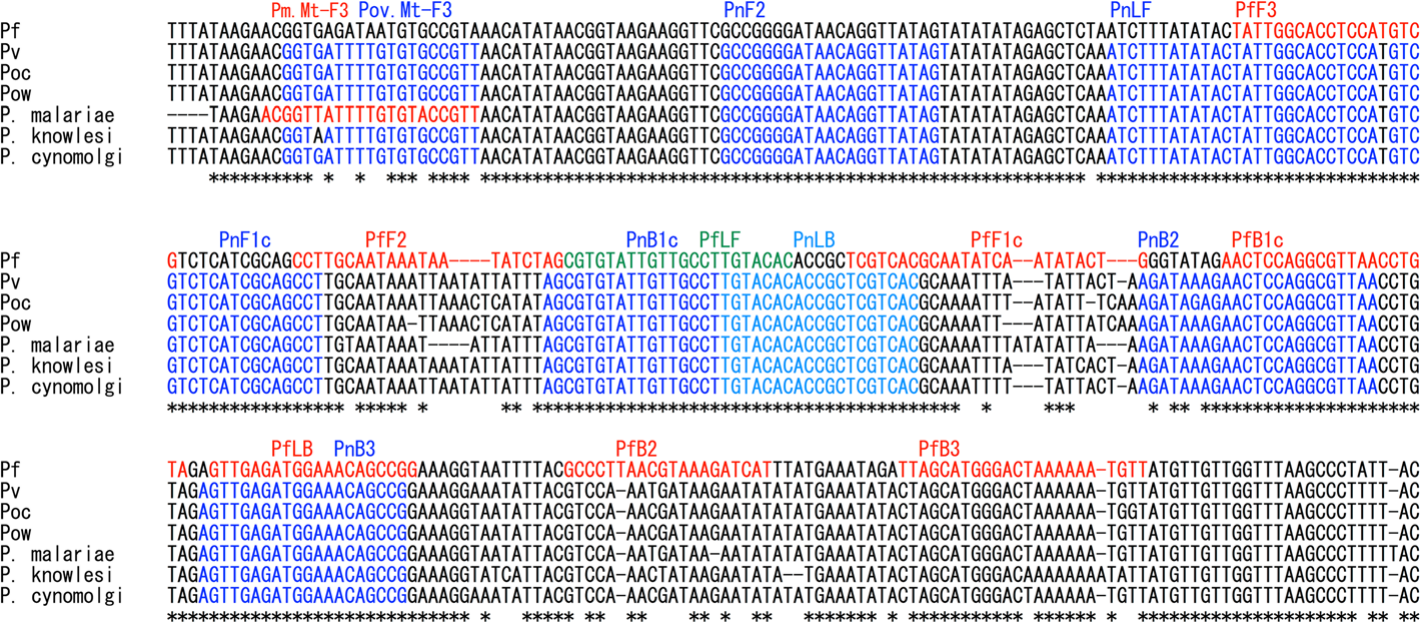
LAMP primers designed for Pf and non-Pf malaria detection. Primer sets for Pf and non-Pf malaria are indicated in *P. falciparum* (Pf), *P. vivax* (Pv), *P. ovale curtisi* (Poc), *P. ovale wallikeri* (Pow), *P. malariae* (Pm), *P. knowlesi* and *P. cynomolgi*

LAMP primer sets were used to detect a part of mitochondrial DNA (mtDNA) of human-infective *Plasmodium* species. The LAMP primer sets consisting of F3 (forward outer primer), B3 (backward outer primer), FIP (forward inner primer), BIP (backward inner primer), LF (loop forward primer), and LB (loop backward primer) were designed as previously described by Notomi et al. [4] and are listed in Table 1. The LAMP primer set targeting mtDNA for non-*P. falciparum* species (*P. malariae*, *P. ovale curtisi*, *P. ovale wallikeri* and *P. vivax*) were designed in this study using online PCR primer design tool Primer3 [6, 7]. The sequences of the same target region are also conserved in *P. knowlesi* and *P. cynomolgi* (Fig. 1). We also modified *P. falciparum* mtDNA detection primer sets reported in a previous literature [8]. After the primer sets were evaluated by real-time LAMP using the Rotor-Gene 3000 thermocycler (Corbett Research, Sydney, Australia) to select the best candidate primer sets by monitoring the reaction speed, cross-reactivity, primer multimer formation and optimal amplification time, the optimal temperature of LAMP reaction for the primer sets was determined.

**Table 1:** LAMP and nested PCR primer sequences for Pf and non-Pf used in this study

Primer set	Primer	Target species	Sequence (5'-3')
Non-Pf CZC-LAMP primer set (7 primers)	Non-Pf.Mt-FIP	Pv, Poc, Pow, Pm	AGGCTGCGATGAGACGACGCCGGGGATAACAGGTTATAGT
	Non-Pf.Mt-BIP	Pv, Poc, Pow, Pm	AGCGTGTATTGTTGCCTTGTACTTAACGCCTGGAGTTCTTTATCTT
	Non-Pf.Mt-LF	Pv, Poc, Pow, Pm	TGGAGGTGCCAATAGTATATAAAGAT
	Non-Pf.Mt-LB	Pv, Poc, Pow, Pm	TGTACACACCGCTCGTCAC
	Pov.Mt-F3	Pv, Poc, Pow	GGTGATTTTGTGTGCCGTT
	Pm.Mt-F3	Pm	ACGGTTATTTTGTGTACCGTT
	Non-Pf.Mt-B3	Pv, Poc, Pow, Pm	CGGCTGTTTCCATCTCAACT
Pf CZC-LAMP primer set (6 primers)	Pf.newMt-FIP	Pf	CAGTATATTGATATTGCGTGACGACCTTGCAATAAATAATATCTAGCGTGT
	P.f.Mt-BIP^a^	Pf	AACTCCAGGCGTTAACCTGTAATGATCTTTACGTTAAGGGC
	Pf.newMt-LF	Pf	GTGTACAAGGCAACAATACACG
	P.f.Mt-LB^a^	Pf	GTTGAGATGGAAACAGCCGG
	Pf.newMt-F3	Pf	TATTGGCACCTCCATGTCG
	P.f.Mt-B3^a^	Pf	AACATTTTTTAGTCCCATGCTAA
Non-Pf nested PCR primers	PnfMt-Fout	Pv, Poc, Pow, Pm	TGCTGTCATACATGATGCACTT
	PnfMt-Rout	Pv, Poc, Pow, Pm	ATGTAGTTTCCTCACAGCTTTATTCA
	PnfMt-Fin	Pv, Poc, Pow, Pm	TGCTGTCATACATGATGCACTT
	PnfMt-Rin	Pv, Poc, Pow, Pm	ATAACATTTTTTAGTCCCATGCTAGTA

^a^The published LAMP primer sequence (PfMt869) [8] was used

*Abbreviations*: *Pv P. vivax*, *Pow P. ovale wallikeri*, *Poc P. ovale curtisi*, *Pm P. malariae*, *Pf P. falciparum*

### LAMP reaction

The preparation of dry LAMP (CZC-LAMP) reagent was previously described [5]. Briefly, 3.0 μmol of trehalose, 35 nmol each of dNTPs mix and 8 U of *Bst*2.0 WS DNA polymerase were mixed and dried on the lid of a 0.2 ml microtube. Colori-Fluorometric Indicator (CFI; 3 nmol hydroxyl-naphtol blue with 3.5 nl GelGreen™ (10,000× Sol), 3.2 pmol of FIP and BIP, 0.4 pmol of F3 and B3, and 1.6 pmol of LF and LB primers were mixed and dried to prepare a dry LAMP tube.

When genomic DNA purified from the blood from malaria infected patients were used as a reaction template, 1 μl of extracted DNA solution plus 24 μl of 1× LAMP reaction buffer (20 mM Tris-HCl [pH 8.8], 10 mM KCl, and 7 mM MgSO_4_ and 0.1% TritonX-100 in DDW) were added to make a 25 μl reaction mix, which was added to the LAMP reaction tube. The tubes were turned upside down several times, so that the dried reagents were completely reconstituted. For the direct blood LAMP method, 1 μl of anticoagulated patient blood was mixed with 24 μl of 1× LAMP buffer, which had been placed in a dry LAMP tube. After the reaction tubes were incubated at 60 °C for 40 min for *P. falciparum* and 60 min for non-*P. falciparum* detection, amplified DNA products were visualized using GelGreen™ contained in the CFI using battery-driven hand-made LED illuminator that was designed for suitable field use [5]. Positive samples exhibited a bright fluorescent green colour under a transilluminator, and two independent observers recorded the result.

### *Plasmodium* nested PCR

Nested PCR for *P. falciparum* and non*-P. falciparum* SSUrRNA was performed according to the published methods [9]. Nested PCR detection of non-*P. falciparum* infection was double-checked by mtDNA nested PCR primers newly designed in this study by using Primer 3 online program (Table 1). After 25 cycles of 1st PCR (denaturing at 98 °C for 10 s, annealing at 64 °C for 30 s and extension at 72 °C for 50 s), 0.5 μl of 1st PCR product was used for 2nd PCR template which was subjected to 25 cycles of the same program as 1st PCR. All PCR reactions were carried out with ExTaq Hot Start Version (Takara-Bio, Shiga, Japan) in a volume of 20 μl. Amplicons of the SSUrRNA and mtDNA nested PCR were sequenced by ABI 3130 capillary sequencer to identify the species of non-*P. falciparum*. In case of detection of mixed wave patterns in the DNA sequencing, the amplicon was cloned into pGEM-T Easy vector (Promega, USA), and then several clones were sequenced. Before the final confirmation of results, all negative samples of first screening were re-examined by nested PCR.

### Immunochromatographic test (ICT) for malaria

As a malaria conventional RDT that immunologically detects and differentiate *P. falciparum* and non-*P. falciparum* species, Hexagon Malaria Combi™ test (Human Gesellschaft für Biochemica und Diagnostica, Wiesbaden, Germany) was used in accordance with the manufacturers’ instructions. The results were read at 15 min after the first diluent drop had been applied into the sample window. Even a faint color band was read as positive. Two independent observers confirm and record the result.

### Field studies

Malaria diagnoses, ICT and malaria CZC-LAMP were performed for 35 voluntary participated residents at Chinshimbwe in the eastern Zambia, (14°3'2.12"S, 30°58'48.15"E) on August 15th and 61 residents at Shikabeta (14°57'12.41"S, 29°49'54.54"E) in the east central Zambia, on August 20th 2013 (Additional file 1: Figure S1). After a brief interview and signing an informed consent form, 2 ml of venous blood were collected in a heparin-containing BD Vacutainer tubes (Becton, Dickinson and Company, USA). Hexagon malaria combi test, and CZC-LAMP tests were performed within 10 min after blood collection as described above. Blood samples were kept at 4 °C for 3 to 4 days during transportation to Lusaka, and frozen at -80 °C in the laboratory at the University of Zambia, Lusaka. About 200 μl of each blood sample was used for DNA extraction by using QuickGene DNA whole blood kit (FujiFilm, JAPAN). These DNA samples were used for the nested PCR analysis.

Human blood samples collected at Mwanya in the eastern Zambia (12°72'82.93"S, 32°24'88.54"E) on October 24th, 2012 were also used to establish our LAMP system.

### Data management and statistical analysis

LAMP, ICT and PCR data were entered in Microsoft Office Excel 2011. Nested-PCR results were used as reference tests. Sensitivity was estimated as the number of both LAMP and nested-PCR positives divided by the number of nested-PCR positives. Specificity was estimated as the number of both LAMP and nested-PCR positives divided by the total number of LAMP positives. Binomial exact 95% CIs were calculated for sensitivities and specificities.

## Results

### Malaria nested PCR diagnosis for extracted blood DNA

Prior to develop malaria CZC-LAMP, extracted DNAs from human blood samples collected from Mwanya in eastern Zambia were analyzed by malaria nested PCR (Table 2). Based on this result on 74 asymptomatic Mwanya individuals, 42 were parasite DNA-positive while 32 were negative. Among the positive samples, 21 were *P. falciparum* (Pf)-single infection, 3 were non-*P. falciparum* (non-Pf) single infection and 18 were mixed infection with Pf /non-Pf. The details of non-Pf infections were single infection with *P. malariae* (Pm; 13 cases), *P. ovale curtisi* (Poc; 4), *P. ovale wallikeri* (Pow; 2), and other two cases were Pm/Poc mixed infections. When the nested PCR results were used as reference, the sensitivities of ICT were 48.7% (95% CI: 32.5–64.9) for Pf and 14.3% (95% CI: 0–30.2) for non-Pf, the specificities were 95.0% (95% CI: 84.8–100) for Pf and 60.0% (95% CI: 0–100) for non-Pf. The integrated sensitivity and specificity of malaria ICT for Pf and non-Pf were 47.6 and 95.2%, respectively.

**Table 2:** Results of malaria diagnostic tests for archived extracted DNA of blood samples collected from Mwanya, eastern Zambia

ID	ICT	Non-Pf CZC-LAMP	Non-Pf Nested PCR	PfMt869 LAMP [8]	Pf CZC-LAMP	Pf Nested PCR
1	–	–	–	+	+	+
7	–	–	–	+	+	+
9	F	–	Pow	+	+	+
10	–	–	–	–	–	+
11	–	+	Pow	–	–	–
13	–	–	–	+	+	+
14	F	–	–	+	+	+
15	F	–	–	+	+	+
17	F	+	Pm	+	+	+
18	–	–	Pm + Poc	+	+	+
19	–	–	–	+	+	+
20	–	–	–	+	+	+
23	–	–	–	+	+	+
27	F, NF	+	Poc	+	+	+
29	F	–	–	+	+	+
30	F	+	Poc	+	+	+
32	–	+	Pm	+	+	+
34	–	–	Pm + Poc	+	+	+
40	F, NF	–	–	+	+	+
41	–	–	–	–	–	+
42	–	+	Pm	–	–	+
43	–	–	–	+	+	+
44	–	+	Poc	–	–	–
45	F	–	–	+	+	+
50	NF	+	Pm	–	–	–
52	F	+	Pm	+	+	+
54	–	–	–	–	–	+
55	F	+	Pm	+	+	+
58	F	+	Pm	+	+	+
61	F, NF	+	Pm	+	+	+
63	F	+	Poc	+	+	+
64	F	+	Pm	+	+	+
65	F	–	–	+	+	+
66	–	+	Pm	+	+	+
71	–	–	–	–	+	+
73	F, NF	–	–	–	–	–
75	–	–	–	+	+	+
80	–	–	–	+	+	–
85	–	+	Pm	–	+	+
87	F	+	Pm	+	+	+
89	–	–	–	+	+	+
91	F	–	–	+	+	+
95	–	+	Pm	+	+	+
103	F	–	–	+	+	+

*Abbreviations*: *F P. falciparum* positive in ICT, *NF* non-*P. falciparum* positive in ICT, *Pow P. ovale wallikeri*, *Poc P. ovale curtisi*, *Pm P. malariae*

### Development and evaluation of Pf and non-Pf detecting LAMP primers using extracted blood DNA samples

To differentiate Pf from non-Pf, we designed several LAMP primer sets so that mtDNAs of non-Pf species can be detected universally without cross-reaction with Pf. The extracted DNA samples of non-Pf single infection (ID #11 and 50 in Table 2) were used as template for screening and selection. At the same time, those of Pf single infection (ID #1 and 7 in Table 2) were used as negative control to exclude Pf cross-reactive primers. After the real time LAMP and melting curve analyses of those DNA samples, the best primer set was selected (Fig. 1). In the same way, Pf-specific LAMP primer set was also selected. Because there is a published LAMP primer set which detects Pf mtDNA specifically [8], we also tested these six primers as candidates, while half of them were replaced by newly designed primers to improve sensitivity and specificity (Table 1).

The performances of non-Pf and Pf primer sets were evaluated by using extracted DNA samples described above (Table 2). Of the 74 samples, non-Pf LAMP specifically detected 18 non-Pf PCR positive samples without cross-reaction to Pf or nonspecific reaction to malaria-free DNA samples. Although non-Pf LAMP detected Pm and Po equally, these two species could not be detected in three of non-Pf PCR-positive samples (ID #9, 18 and 34). On the other hand, Pf LAMP reacted to 35 Pf PCR-positive samples and to one (ID #80) malaria PCR-negative one, but missed four Pf PCR-positive samples (ID #10, 41, 42 and 54). The existing LAMP primer set (PfMt869) [8] was also used for comparison. LAMP with PfMt869 primer sets detected 33 Pf PCR positive samples and also detected the same malaria PCR-negative sample that was positive by CZC-Pf LAMP (ID #80 in Table 2). If the nested PCR results were used as a standard, sensitivities were 85.7% for non-Pf LAMP and 89.7% for Pf LAMP; specificities were 100% for non-Pf LAMP and 97.2% for Pf LAMP. Nested PCR-positive and ICT-negative samples were supposed to be infections with very low parasitemia. The sensitivities of three LAMP primer sets for those of low density samples were 83.3% (non-Pf), 80.0% (Pf) and 70.0% (PfMt869) (Table 3). All nested PCR-positive and ICT-positive which were supposed to be high parasitemic samples were detected by non-Pf, Pf and PfMt869 LAMP primer sets with 100% of sensitivity.

**Table 3:** Diagnostic accuracy of malaria LAMPs for archived extracted DNA of blood samples collected from Mwanya, eastern Zambia

		Pf CZC-LAMP	PfMt869-LAMP^a^	non-Pf CZC-LAMP
		% (95% CI)	% (95% CI)	% (95% CI)
Total	Sensitivity	89.7 (79.9–99.5)	84.6 (72.9–96.3)	85.7 (69.9–100)
	Specificity	97.2 (91.7–100)	97.1 (91.2–100)	100
ICT positive	Sensitivity	100	100	100
	Specificity	100	100	100
ICT negative	Sensitivity	80.0 (61.3–98.7)	70.0 (48.6–91.4)	83.3 (64.8–100)
	Specificity	94.1 (82.0–100)	93.3 (79.5–100)	100

^a^The published LAMP primer sequence (PfMt869) [8] was used

### Evaluation of malaria CZC-LAMP in field surveillance at the malaria endemic areas in Zambia

**Fig. 2:**
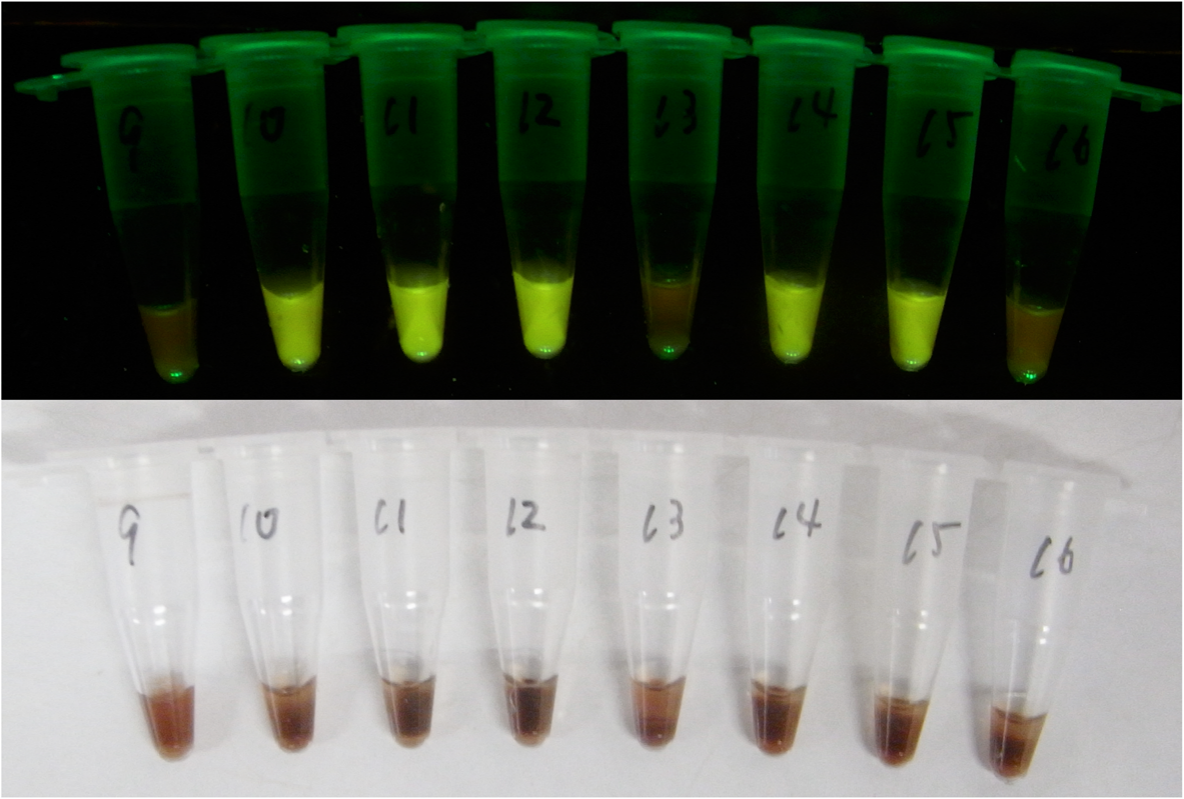
Visual determination of positive and negative samples. Examples of malaria CZC-LAMP tubes after the reaction. It is not possible to determine positive or negative under natural light due to the interference by hemoglobin (*lower*). However, positive samples emit green fluorescence under 505 nm blue-green light (*upper*)

Next, we evaluated the performance of Pf and non-Pf malaria CZC-LAMP in the malaria endemic areas where we used 1 μl of anticoagulated unprocessed blood for each kit instead of using purified DNA or other processed blood materials. For the diagnosis, ICT was also performed at the same time. Representative visual appearance of test tubes after Pf CZC-LAMP reaction is shown in Fig. 2, and the results are summarized in Tables 4 and 5.

**Table 4:** Results of malaria CZC-LAMP for blood samples collected from Chinshimbwe, eastern Zambia

ID	ICT	Non-Pf CZC-LAMP	Non-Pf Nested PCR	Pf CZC-LAMP	Pf Nested PCR
C01	–	+	Pm	+	+
C04	F	+	Pm	+	+
C05	–	–	–	+	+
C06	F	–	–	+	+
C07	–	–	–	+	+
C09	–	–	–	+	+
C10	F, NF	–	–	+	+
C12	F	–	–	+	+
C14	–	–	–	+	+
C15	F	+	Poc	+	+
C16	–	–	–	+	+
C17	F	–	–	+	+
C19	F	–	–	+	+
C22	F	+	Pm	+	+
C23	F	–	–	+	+
C24	–	+	Pm	+	+
C25	–	–	–	+	+
C26	–	+	Pm	+	+
C27	F	–	–	+	+
C30	F, NF	+	Pm	+	+
C31	F	–	–	–	–
C32	F	–	–	+	+
C33	F	–	–	–	–
C35	–	+	Pow	+	+

*Abbreviations*: *F P. falciparum* positive in ICT, *NF* non-*P. falciparum* positive in ICT, *Pow P. ovale wallikeri*, *Poc P. ovale curtisi*, *Pm P. malaria*

**Table 5:** Results of malaria CZC-LAMP for blood samples collected from Shikabeta, eastern Zambia

ID	ICT	Non-Pf CZC-LAMP	Nested PCR non-Pf	Pf CZC-LAMP	Nested PCR Pf
S03	–	–	–	+	+
S06	–	–	–	+	+
S09	–	+	Poc	–	–
S10	F, NF	+	Pm	+	+
S11	F, NF	+	Pm	+	+
S12	F	+	Pm	+	+
S14	F	+	Pm	+	+
S15	F	+	Pm + Poc	+	+
S18	–	–	–	+	+
S19	F	+	Pm	–	–
S20	–	+	Pm	–	–
S22	–	+	Poc	+	+
S23	F, NF	+	Pm	+	+
S24	–	–	–	+	+
S25	F	+	Pm	+	+
S26	F	–	–	–	–
S27	–	+	Poc	–	–
S29	–	+	Pm	+	+
S31	F, NF	+	Pv	+	+
S32	F	+	Pm	+	+
S33	–	+	Pm	–	–
S35	F, NF	+	Pm	+	+
S36	F, NF	+	Pm	+	+
S37	F, NF	+	Pm	+	+
S38	F	–	–	+	+
S39	–	+	Pow	–	–
S40	–	–	–	+	+
S41	F	–	–	–	–
S42	F, NF	+	Pm	+	+
S43	F	+	Pow	+	+
S44	NF	+	Pm	–	+
S47	–	–	–	+	+
S48	F	+	Pm	+	+
S50	–	–	Poc	–	–
S51	F	–	Pm	+	+
S52	F, NF	+	Poc	+	+
S54	F, NF	+	Pm	+	+
S56	–	–	–	+	–
S57	F	–	–	–	–
S59	–	–	Pm	+	+
S61	F, NF	+	Pm	+	+

*Abbreviations*: *F P. falciparum* positive in ICT, *NF* non-*P. falciparum* positive in ICT, *Pow P. ovale wallikeri*, *Poc P. ovale curtisi*, *Pm P. malariae*

Of the 35 blood samples in Chinshimbwe, 8 were positive for non-Pf CZC-LAMP and 22 were positive for Pf CZC-LAMP, while ICT detected 2 non-Pf and 14 Pf. At this sampling site, nested PCR results which had been done later at the laboratory in Lusaka were exactly matched to those obtained by both non-Pf and Pf malaria CZC-LAMPs, resulting in 100% of the sensitivities and specificities. In case of ICT, we observed false positive cases in 2 Pf and 1 non-Pf samples. The sensitivities of ICT were 13% and 55%, and the specificities were 50% and 80% for non-Pf and Pf, respectively.

Of the 61 blood samples collected from Shikabeta area, nested PCR detected 30 non-Pf-positive cases (21 Pm, 5 Poc, 2 Pow, 1 *P. vivax* [Pv] single infections and 1 Pm/Poc mixed infection). Non-Pf CZC-LAMP detected 27 of the 30 PCR-positive cases, 3 false negatives but no false positive samples. In addition, 30 Pf-positive samples were detected by nested PCR. CZC-LAMP detected 29 nested PCR-positive samples and 1 false positive sample when the Pf nested PCR results were used as a reference. The sensitivities and specificities of non-Pf/Pf CZC-LAMP in Shikabeta were 90%/96.7% and 100%/96.7%, respectively. On the other hand, the sensitivities and specificities of non-Pf/Pf ICT were 40%/67% and 100%/87%, respectively.

Overall sensitivities and specificities of non-Pf CZC-LAMP obtained during this study were 92.1 and 100%; and Pf CZC-LAMP were 98.1 and 98.1%, respectively (Table 6). On the other hand, the sensitivities and specificities of ICT were 34.2% (95% CI: 18.6–49.8) and 92.9% (95% CI: 78.0–100) for non-Pf, 61.5% (95% CI: 47.9–75.1) and 86.5% (95% CI: 75.1–97.9) for Pf, respectively. In addition, although non-Pf CZC-LAMP reacted to four non-Pf malaria species (Pm, Poc, Pow and Pv), cross-reaction between non-Pf and Pf was not observed at all. As is the case with purified DNA samples, non-Pf and also Pf CZC-LAMPs detected all high-parasitemic samples which were both nested PCR- and ICT-positive. In the case of low density conditions, the sensitivities of non-Pf and Pf CZC-LAMP were 88.0 and 95.0%, respectively (Table 6).

**Table 6:** Diagnostic accuracy of malaria CZC-LAMP for blood samples collected from Chinshimbwe and Shikabeta, eastern Zambia

		Pf CZC-LAMP	Non-Pf CZC-LAMP
		% (95% CI)	% (95% CI)
Total	Sensitivity	98.1 (94.3–100)	92.1 (83.3–100)
	Specificity	98.1 (94.3–100)	100
ICT positive	Sensitivity	100	100
	Specificity	100	100
ICT negative	Sensitivity	95.0 (84.8–100)	88.0 (74.6–100)
	Specificity	95.0 (84.8–100)	100

## Discussion

In this study, we conducted field surveillance studies of malaria in the remote rural parts of the eastern Zambia using a field-applicable molecular diagnosis system, CZC-LAMP. We demonstrated that Pf and non-Pf CZC-LAMPs were substantially superior in terms of sensitivity and specificity to the conventional ICT. Not only did both Pf and non-Pf CZC-LAMP perfectly detected parasite DNA from moderate-density parasitemia samples which were both ICT- and nested PCR-positive, but non-Pf CZC-LAMP improved the sensitivity of non-Pf malaria diagnosis (34.2% by ICT to 92.1%) without sacrificing specificity. In addition, Pf CZC-LAMP exhibited better sensitivity than existing Pf LAMP and was thus almost as sensitive as nested PCR when blood samples were directly subjected to the analysis without any DNA extraction or purification steps.

Our initial aim was to develop a field-applicable simplified molecular diagnosis method, but we have also achieved increased sensitivity with both Pf and non-Pf CZC-LAMP for low-parasitemia samples when the test samples switched from purified DNA to anticoagulated otherwise unprocessed fresh blood (83.3 to 88% for non-Pf and 80 to 95% for Pf). Therefore, the LAMP method in which unprocessed blood is used exhibits a superior sensitivity to that of the existing LAMP method which employs extracted DNA from the blood. This minimization of sample preparation process has advantage for the point-of-care test under resource poor settings.

ICT is most commonly used Malaria RDTs showing high sensitivities and specificities, especially when parasite burden is high in sick patient. It has been demonstrated that Hexagon rapid diagnostic test showed 100% sensitivity both for Pf and non-Pf, if > 5,000 parasite/μl blood were used. But this sensitivity was dramatically dropped when low-density patient blood showing < 500 parasite/μl were used, showing 50 and 0% sensitivity for Pf and non-Pf [10]. As such, although ICT is useful in terms of simple operation and sensitivity for high parasitemia patient, more sensitive and specific alternative diagnostics must be developed for controlling malaria in endemic areas.

For the purpose of malaria DNA detection, commercial malaria LAMP kits to detect Pf and pan-plasmodium species (Loopamp™ MALARIA Pan/Pf Detection Kit, Eiken Chemical Company, Japan) are already available. Although these kits also demonstrated excellent performance in malaria detection, even at low-density parasitemia, comparable to nested PCR in minimally equipped laboratories [11–13], our Pf/non-Pf CZC-LAMP have advantages over these kits in the following points. First, the CZC-LAMPs use a minute amount of blood from the patient which can be introduced into the reaction without any process. CZC-LAMP require neither DNA extraction by PURE method nor blood boiling and spin down steps, and are as convenient as conventional ICT using less than 5 μl of blood collected. In addition, direct blood method may contribute to sensitization of LAMP reaction as mentioned above. Secondly, CZC-LAMPs are important to determine whether non-Pf malaria species are in a mixed infection with Pf or not. This suggests that rapid and accurate point-of-care diagnoses of monolithic Pf or non-Pf as well as Pf/non-Pf mixed infection, are possible, facilitating for the immediate and effective medication of the affected patients. The other advantages of CZC-LAMP over existing LAMP were already described in our previous report [5].

Recent molecular epidemiological studies of malaria detection and species discrimination by nested PCR in sub-Saharan Africa have shown that there exist mixed infections of low-density non-Pf species other than Pv with Pf much more than previously considered [14–16]. In Zambia, the situation of mixed infections with low-density Pf and non-Pf had largely remained unexplored until the recent study using stored samples from the children under 6 years old and nested PCR was published [17]. In the present study, we report the detection of many cases of mixed infections of low-density Pf and non-Pf (mainly Pf + Pm and to a lesser extent Pf + Po), and also a few non-Pf monolithic infections at sampling points in the eastern Zambia (Additional file 2: Table S1). Artemisinin used as first-line medicine in several sub-Saharan African counties fails to eliminate Pm, Po and also low-density Pf [18]. These findings suggest that there is a need for continuous monitoring of non-Pf infections and low-density Pf prevalence for effective treatment to malaria species. Our Pf and non-Pf CZC-LAMPs are expected to be extremely useful in the diagnosis or surveillance studies of malaria in sub-Saharan African countries. CZC-LAMPs are also useful means to monitor and confirm the negative transmission in the area where malaria elimination is almost achieved. Because malaria CZC-LAMP is relatively cost-effective and easy for operation, this can be applied for active and massive vector surveillance. Therefore, application of our malaria CZC-LAMP to mosquito lysate or extracted DNA is required in future work.

In conclusion, these Pf and non-Pf CZC-LAMPs showed higher sensitivity/specificity compared to conventional ICT. As for the new RDT, CZC-LAMP will provide new paradigm of malaria infection, low-density chronic infection and transmission. Therefore, these diagnosis tools will not only elucidate the full picture of epidemiology of malaria but are also expected to contribute to reduction or elimination of malaria infections from endemic areas in sub-Saharan Africa.

## Conclusions

Recent studies have indicated that low-density chronic infection of malaria in asymptomatic patient, undetectable by conventional RDTs, is the next problem for malaria infection control. Using human blood DNA samples collected in malaria endemic area in Zambia, we have developed malaria CZC-LAMP kits, new simple and sensitive RDT, for falciparum malaria and non-falciparum (four species) malaria, respectively. These kits detected low-density, even falciparum/non-falciparum mixed, infection with a high degree of accuracy in blood samples collected in malaria endemic areas. This system will elucidate the ecological geography of chronic malaria infection especially for *P. falciparum* and *P. malariae*, both of them do not have dormant liver stage, and may contribute to complete elimination effort of malaria.

## Additional files

Additional file 1: Figure S1. Samples collection sites in Zambia. Map showing human blood sampling sites in Zambia. The red star indicates the location of the sampling site. Red line: road; Blue line: landside waters; Gray line; boundary line. The map was downloaded from the International Steering Committee for Global Mapping. (TIFF 7390 kb)
Additional file 2: Table S1. Infection prevalence of Malaria species at three sampling sites in Zambia. (DOCX 71 kb)

## Abbreviations

HAT: Human African trypanosomiasis.
ICT: Immunochromatographic test
LAMP: Loop-mediated isothermal amplification
Non-Pf: Non-*Plasmodium falciparum* (*P. vivax P. ovale*, *P. malariae)*
Pf: *Plasmodium falciparum*
Pm: *P. malariae*
Poc: *P. ovale curtisi*
Pow: *P. ovale wallikeri*
Pv: *P. vivax*
RDTs: Rapid diagnostic tests
