# Supplementary material for: Direct detection of falciparum and non-falciparum malaria DNA from a drop of blood with high sensitivity by the dried-LAMP system
Source: Parasit Vectors. 2017 Jan 13;10:26. doi: 10.1186/s13071-016-1949-8 (PMC5237333; doi:10.1186/s13071-016-1949-8)
Supplement: Additional file 2: Table S1. — Infection prevalence of Malaria species at three sampling sites in Zambia. (DOCX 71 kb) [file 13071_2016_1949_MOESM2_ESM.docx]

|  | **Total** | | **Mwanya** | | **ChinShimbwe** | | **Shikabeta** | |
| --- | --- | --- | --- | --- | --- | --- | --- | --- |
| **Nested-PCR positive** | # | % | # | % | # | % | # | % |
| **Pf** | 42 | 24.7% | 21 | 28.4% | 14 | 40.0% | 6 | 9.8% |
| **Pm** | 6 | 3.5% | 2 | 2.7% | 0 | 0.0% | 4 | 6.6% |
| **Poc** | 4 | 2.4% | 1 | 1.4% | 0 | 0.0% | 3 | 4.9% |
| **Pow** | 2 | 1.2% | 1 | 1.4% | 0 | 0.0% | 1 | 1.6% |
| **Pv** | 0 | 0.0% | 0 | 0.0% | 0 | 0.0% | 0 | 0.0% |
| **Pf + Pm** | 34 | 20.0% | 11 | 14.9% | 6 | 17.1% | 17 | 27.9% |
| **Pf + Poc** | 6 | 3.5% | 3 | 4.1% | 1 | 2.9% | 2 | 3.3% |
| **Pf + Pow** | 3 | 1.8% | 1 | 1.4% | 1 | 2.9% | 2 | 3.3% |
| **Pf + Pv** | 1 | 0.6% | 0 | 0.0% | 0 | 0.0% | 1 | 1.6% |
| **Pf + Pm + Poc** | 3 | 1.8% | 2 | 2.7% | 0 | 0.0% | 1 | 1.6% |
| **total** | **101** | **59.4%** | **42** | **56.8%** | **22** | **62.9%** | **37** | **60.7%** |

**Table S1: Infection prevalence of Malaria species at three sampling sites in Zambia.**
